# Flow in the time of COVID-19: Findings from China

**DOI:** 10.1371/journal.pone.0242043

**Published:** 2020-11-11

**Authors:** Kate Sweeny, Kyla Rankin, Xiaorong Cheng, Lulu Hou, Fangfang Long, Yao Meng, Lilian Azer, Renlai Zhou, Weiwei Zhang

**Affiliations:** 1 Department of Psychology, University of California, Riverside, California, United States of America; 2 School of Psychology, Central China Normal University, Wuhan, China; 3 Department of Psychology, Nanjing University, Nanjing, China; University of Connecticut, UNITED STATES

## Abstract

In February 2020, the Novel Coronavirus (COVID-19) was raging in Wuhan, China and quickly spreading to the rest of the world. This period was fraught with uncertainty for those in the affected areas. The present investigation examined the role of two potential coping resources during this stressful period of uncertainty: flow and mindfulness. Participants in Wuhan and other major cities affected by COVID-19 (*N* = 5115) completed an online survey assessing subjective experiences of flow, mindfulness, and well-being. Longer quarantine was associated with poorer well-being; flow and mindfulness were associated with better well-being on some measures. However, flow—but not mindfulness—moderated the link between quarantine length and well-being, such that people who experienced high levels flow showed little or no association between quarantine length and poorer well-being. These findings suggest that experiencing flow (typically by engaging in flow-inducing activities) may be a particularly effective way to protect against potentially deleterious effects of a period of quarantine.

## Introduction

According to the World Health Organization (WHO), nearly 36.3 million people are currently confirmed to have Novel Coronavirus (2019-nCoV, or COVID-19) at the time of writing in October 2020. COVID-19, which is deceptively similar to pneumonia and influenza, has impacted healthcare, shifted the political climate, and created a financial crisis. New areas of the world report cases daily, some level of quarantine or social distancing is recommended (if not required) where the virus is present, and on March 11, 2020 the WHO officially identified COVID-19 as a pandemic. Social distancing efforts seek to decrease the number of people in contact with one another with the goal of minimizing the spread of infection. As a more drastic measure, quarantine involves complete isolation, with recommendations directing people testing positive or showing symptoms of COVID-19 to quarantine for 14 days. The present investigation seeks to identify effective strategies for managing distress during this stressful period of worldwide uncertainty, particularly among those facing the psychological challenges of quarantine conditions in Wuhan, China and other affected Chinese cities in February 2020.

### COVID-19 in Wuhan, China

The first case of COVID-19 was detected in Wuhan, China and reported to the WHO China Country Office on December 31, 2019. At the time, the nature, cause, and means of transmission of COVID-19 remained unclear. With the first exported cases reported in Thailand, Japan, and South Korea in mid-January, the Chinese government chose to take action by imposing an emergency lockdown beginning on January 23, 2020, requiring people to remain in the area and self-quarantine. In response to the quickly-spreading virus, many governments around the world followed by recommending, some even mandating, social distancing or quarantine measures.

The spread of this virus has created massive disruption in people’s lives and enormous amounts of uncertainty: Schools are closed or transitioned online, jobs are either substantially reimagined or eliminated altogether, people are testing positive for the virus without showing symptoms, and knowing when the containment of COVID-19 will bring normalcy is impossible. While people wait to see how the virus progresses and when vaccines will become available, physical distancing and self-quarantining are encouraged, if not required, for many.

Research assessing the psychological effects of quarantine-like conditions during previous virus outbreaks (e.g., Severe Acute Respiratory Syndrome, Ebola Virus Disease, and Influenza A virus subtype H1N1) identified several key sources of distress: fear of infection for self and others, inadequate information from health authorities, feelings of frustration and boredom, and, most relevant to the current investigation, quarantine length [1]. Specifically, longer quarantine durations predicted greater post-traumatic stress disorder symptomology [2, 3] and greater emotional exhaustion and distress in healthcare workers [4], and the current lockdown in France for COVID-19 was associated with slowing down of time, seemingly driven by boredom and sadness [5]. Given the negative consequences of increased quarantine length, how can people hold their rising distress at bay in ways that reduce boredom and frustration as quarantine conditions persist and concerns about the impact of the virus grow?

### Harnessing flow and mindfulness while coping with uncertainty

Two promising candidates for effective coping during the COVID-19 pandemic are flow and mindfulness. Flow is a state in which people become absorbed in an enjoyable activity, such that they become blind to their external environment and unconcerned about the self or the passage of time [6]. To reach a flow state, people must engage in an intrinsically rewarding activity that is just challenging enough to match one’s skill level and that provides clear goals and feedback. A large body of evidence links flow to greater well-being in a variety of domains, including work [7, 8], sports [9], collective gatherings and rituals [10], and general leisure activities [11]. Most relevant to this study, flow is effective for boosting emotional well-being during stressful periods of uncertainty [12]. Notably, researchers have documented the benefits of flow across a number of cultures and countries, including Japan [13, 14], Czech Republic [15], Hong Kong [16], Sweden [17], and Italy [18].

In contrast to flow states, mindfulness is a state of being aware of and attentive to one’s current internal and external experience, focusing on the present moment and observing without judgment [19]. Traditionally a Buddhist teaching, mindfulness has become an asset to medicine, healthcare, and psychology [20–24]. As with flow, a large body of evidence links mindfulness to greater well-being, including lower depression and anxiety [21], less psychological distress and invasive negative thoughts [25], and more positive thinking [26]. A meta-analysis of 163 studies (*d* = .28) confirmed that practicing mindfulness meditation is associated with greater well-being on a number of metrics [27]. Most relevant to the current investigation study, mindfulness promotes effective coping during periods of acute uncertainty [28].

Flow and mindfulness are similar in that they both involve focused attention on the present moment. They are also both accessible via active engagement in specific activities—that is, one does not have to be a particular kind of person to experience flow or mindfulness [e.g., flow: 29, 30; mindfulness: 31, 32]. However, they differ in important ways. Most notably, flow reduces self-awareness and awareness of one’s external environment, whereas mindfulness draws one’s attention to internal and external experiences [6, 24, 33]. In fact, experimental work has shown that flow and mindfulness are antithetical. When people practice mindfulness prior to engaging in a flow-inducing activity, they report feeling less absorbed in the activity [34].

The current investigation compares associations between flow and mindfulness on the one hand and well-being on the other during a period of stressful uncertainty, specifically the rise of COVID-19 in Wuhan and other affected cities in China. We hypothesized that a longer quarantine would be associated with poorer well-being and that flow and mindfulness would be associated with greater well-being overall during this period. We further hypothesized that flow and mindfulness may play different roles in mitigating the deleterious effects of quarantine length on well-being. Because flow states reduce self-awareness and speed the apparent passage of time (i.e., time “flies by”), we anticipated that it may be particularly effective for reducing the impact of longer quarantines on well-being. Given that our study assessed self-reported flow and mindfulness (i.e., no experimental intervention), our specific hypothesis is that flow will consistently moderate associations between quarantine status and various measures of well-being. We did not have a strong *a priori* prediction about the moderating role of mindfulness.

## Method

### Participants and procedure

This study (#NJUPSY202002002) was approved by the Ethics Committee of Nanjing University, and consent was obtained via an online document (participants clicked "continue" if they consented to participate in the survey). Participants in China (*N* = 5115; see Table 1 for demographic information) were recruited online between February 12–19, 2020. We recruited as many participants as possible during the relevant period of time. We did not conduct a power analysis; however, our sample size is more than sufficient for the relevant analyses at nearly any effect size.

**Table 1:** Demographic information.

Sample Characteristics	(*N* = 5115)
% female	72.8%
Mean age	21.36 (*SD* = 4.39)
Education	
Did not complete high school	0.3%
Completed high school only	1.2%
Completed college (2- or 4-year degree)	95.9%
Completed Master’s degree	3.5%
At least 1 sibling	28%
Family income	
Less than $50,000	50.8%
$50,000-$100,000	31.3%
$100,000-$200,000	11.2%
$200,000-$300,000	3.1%
Above $300,000	3.7%

The online survey was hosted on IQEQ (Intelligence Quotient and Emotional Quotient), a dedicated online research platform with a mobile-friendly interface for smartphones and tablets, which was developed by one of the co-authors (R.Z.) at Nanjin University. Participant recruitment was targeted at college students via social media (WeChat platform). No course credit or monetary reward was offered for participants. The survey took participants an average of 9 minutes to complete.

### Measures

Due to the challenging context in which this study was conducted, we were only able to survey our participants once. Thus, our primary measures asked participants to respond based on their experiences “in the past week” in an effort to capture general experiences during that period of time rather than experiences on what could have been an idiosyncratic day. Similarly, we were only able to contact participants remotely (i.e., online), and thus we relied on self-report measures as objective observational measures were infeasible. All scales, originally in English, were translated into Chinese by three co-authors (X.C., R.Z., and W.Z.). Specifically, X.C. and R.Z. translated the scales independently, and W.Z. then verified and combined the translations. The high degree of internal reliability achieved for all of the scales provide reassurance that this translation process was successful.

#### Flow and mindfulness

Flow experienced during the past week was assessed using the five-item Short Flow Scale [6, 12, 35, 36] (“I felt very interested in what I was doing,” “I felt unaware of myself; I was only aware of the tasks at hand,” “I felt very absorbed in what I was doing,” “I felt that there was no separation between me and my behavior,” and “I felt very stimulated and challenged”; 1 = *not at all*, 7 = *very much*; *M* = 4.37, *SD* = 1.13, Cronbach’s *α* = .80). Although this scale has not undergone formal validation, several studies have confirmed its association with positive emotions [12, 36], as well as negative associations with negative emotions and worry during periods of stressful uncertainty [12].

Mindfulness experienced during the past week was assessed using the 12-item Cognitive and Affective Mindfulness Scale-Revised [37] (e.g., “It is easy for me to concentrate on what I am doing,” “I try to notice my thoughts without judging them”; 1 = *rarely/not at all*, 4 = *almost always; M* = 2.50; *SD* = .47, *α* = .75). This measure has undergone extensive validation [37], including in China [38]. The correlation between flow and mindfulness was quite strong, *r*(5115) = .56, *p* < .0001, although not so strong as to suggest that they represent the same construct.

#### Quarantine length

Quarantine length was coded as follows: 1 = 0 days (not yet quarantined; 74% of the sample), 2 = 1–7 days (2%), 3 = 7–14 days (6%), and 4 = more than 14 days (18%). Because the measure was positively skewed, we log-10 transformed it. Although the variable was still skewed after transformation (skewness = 1.16), skewness was notably reduced. Note that quarantine in China was largely mandatory and quite strict, not optional or loosely defined as it is for most people in the United States.

#### Worry and emotions

We assessed worry using three items adapted from the McCaul Brief Worry Scale, standardizing each item before averaging due to differing scaling across items [39] (e.g., “How worried are you about the coronavirus?;” 1 = *not at all*, 5 = *extremely*; *M* = -.0003; *SD* = .85, *α* = .81). Emotions were assessed with the Scale of Positive and Negative Experience [40] (1 = *very rarely or never*, 5 = *very often or always*); specifically, positive emotion experienced during the past week was assessed with six items (e.g., positive, pleasant; *M* = 3.69; *SD* = .79, *α* = .64), as well as negative emotion (e.g., negative, afraid; *M* = 1.93; *SD* = .63, *α* = .88).

#### Depression and anxiety symptoms

We used the Brief Symptom Inventory [41] (0 = *not at all*, 4 = *very much*) to assess depression and anxiety symptoms experienced in the past week, which includes a six-item depression subscale (e.g., feeling blue, feeling hopeless about the future; *M* = .57; *SD* = .61, *α* = .84) and a six-item anxiety subscale (e.g., feeling tense, feeling suddenly scared; *M* = .59; *SD* = .67, *α* = .90).

#### Loneliness

We assessed loneliness during the past week using a three-item Brief Loneliness Measure [42] (“How often have you felt that you lacked companionship?” How often have you felt left out?” “How often have you felt isolated from others?”; 1 = *hardly ever*, 3 = *often*; *M* = 1.37; *SD* = .50, *α* = .78).

#### Health behavior

We assessed health behavior by asking participants how many days over the past week (out of seven days) they engaged in six health-related activities and then summed the three healthy behaviors (engaging in aerobic physical activity for at least 15 minutes, engaging in strengthening exercises, and eating fruits or vegetables; *M* = 4.09; *SD* = 1.45, *α* = .65) and the three unhealthy behaviors (eating junk food, smoking cigarettes/cigars/electronic cigarettes/using chewing tobacco, and having three or more alcoholic beverages; *M* = 1.77; *SD* = .92, *α* = .48).

#### Covariates

In addition to demographic characteristics (gender, age, level of education, sibling status, household income), we measured several uncertainty-relevant covariates, including dispositional optimism, intolerance of uncertainty, and satisfaction with life. We assessed dispositional optimism with the Life Orientation Test-Revised [43] (6 items after removing filler items; e.g., “In uncertain times, I usually expect the best,” “I hardly ever expect things to go my way”; 1 = *strongly disagree*, 5 = *strongly agree*; *M* = 3.80, *SD* = .66, *α* = .80). We assessed intolerance of uncertainty with the 12-item Intolerance of Uncertainty–Short Form [44] (e.g., “Unforeseen events upset me greatly,” “It frustrated me not having all the information I need”; 1 = *not at all characteristic of me*, 5 = *extremely characteristic of me*; *M* = 2.22, *SD* = .66, *α* = .87). Finally, we assessed satisfaction with life using the 5-item Satisfaction with Life Scale [45] (e.g., “In most ways my life is close to my ideal,” “The conditions of my life are excellent”; 1 = *strongly disagree*, 5 = *strongly agree*; *M* = 3.98, *SD* = 1.14, *α* = .83). To provide the most conservative estimate of our effects, we included all available demographic variables, as well as the three individual difference measures just described (the only ones available in the dataset), as covariates in our analyses.

## Results

We tested our hypotheses with multiple regression analyses predicting health and well-being from quarantine length, flow, mindfulness, and the interactions between quarantine length and flow/mindfulness. All variables were grand-mean centered. We controlled for gender, age, education, region, sibling status, family income, dispositional optimism, intolerance of uncertainty, and satisfaction with life in all analyses. Given the large sample size (*N* = 5115), we define statistical significance for our purposes as effects at *p* < .01. Table 2 presents bivariate correlations between our variables.

**Table 2:** Bivariate correlations.

	Mindful-ness	Quarantine length	Worry	Negative emotion	Positive emotion	Depressive symptoms	Anxious symptoms	Loneli-ness	Healthy behaviors	Unhealthy behaviors
Flow	.56*	-.03	-.02	-.16*	.46*	-.22*	-.14*	-.26*	.33*	-.16*
Mindfulness		.02*	-.03	-.18*	.44*	-.19*	-.13*	-.12*	.26*	-.06*
Quarantine length			.13*	.10*	-.04	.14*	.12*	.16*	.03	.10*
Worry				.34*	-.03	.25*	.35*	.19*	.007	.11*
Negative emotion					-.29*	.62*	.66*	.34*	-.13*	.13*
Positive emotion						-.33*	-.24*	-.31*	.31*	-.17*
Depressive symptoms							.73*	.43*	-.17*	.16*
Anxious symptoms								.35*	-.13*	.12*
Loneliness									-.17*	.31*
Healthy behaviors										-.02*

Note

^*^p < .01.

Table 3 presents the full regression results for each measure of health and well-being. Note that quarantine length was a significant predictor of all but one measure (positive emotion). In all but one case (healthy behavior), a longer quarantine was associated with poorer well-being.

**Table 3:** Keys results of multiple regression models.

	Quarantine Length	Flow	Mindfulness	Flow x Quarantine Length	Mindfulness x Quarantine Length
	*β [CI*_*95%*_*]*	*β [CI*_*95%*_*]*	*β [CI*_*95%*_*]*	*β [CI*_*95%*_*]*	*β [CI*_*95%*_*]*
Worry	.10*	.001	-.03	-.04*	.02
	[.07, .13]	[-.03, .03]	[-.06, .003]	[-.07, -.01]	[-.02, .06]
Negative emotion	.06*	.004	-.09*	-.05*	.01
	[.03, .08]	[-.03, .03]	[-.12, -.06]	[-.07, -.02]	[-.02, .05]
Positive emotion	-.02	.17*	.19*	-.004	-.02
	[-.04, .006]	[.14, .19]	[.16, .22]	[-.03, .02]	[-.05, .01]
Depressive symptoms	.09*	-.04*	-.06*	-.06*	.01
	[.07, .11]	[-.06, -.01]	[-.08, -.03]	[-.09, -.04]	[-.01, .04]
Anxious symptoms	.07*	-.004	-.05*	-.06*	.03
	[.04, .09]	[-.03, .02]	[-.08, -.02]	[-.08, -.03]	[.0002, .06]
Loneliness	.12*	-.15*	.06*	-.05*	.01
	[.10, .15]	[-.18, -.12]	[.03, .09]	[-.08, -.02]	[-.02, .05]
Healthy behaviors	.04*	.19*	.06*	-.01	.005
	[.01, .07]	[.16, .22]	[.03, .09]	[-.05, .02]	[-.03, .04]
Unhealthy behaviors	.08*	-.12*	.05*	-.05*	.04
	[.05, .11]	[-.15, -.08]	[.01, .08]	[-.08, -.02]	[.001, .08]

Note

^*^p < .01. Estimates are from models that control for gender, age, sibling status, education, family income, dispositional optimism, intolerance of uncertainty, and satisfaction with life. ^a^1 = 0 days, 2 = 1–7 days, 3 = 7–14 days, 4 = more than 14 days.

### Flow

Looking first at flow’s overall association with well-being, the results were mixed (see Table 3 for key results; full results available on the Open Science Framework along with study materials). Participants who reported greater flow also reported more positive emotion, less severe depressive symptoms, less loneliness, more healthy behaviors, and fewer unhealthy behaviors. However, interactions with quarantine length were more consistent. With the exception of positive emotion and healthy behaviors, flow significantly moderated the relationship between quarantine length and all other measures of well-being. Table 4 presents the associations between quarantine length and well-being at +/-1 SD for flow. As hypothesized, these associations are quite robust for those who reported relatively little flow, whereas they weakened considerable (nearly disappearing in some cases) among those who reported relatively high levels of flow.

**Table 4:** Associations between quarantine length and well-being, modeled at +/-1 SD for flow.

	-1 SD Flow	+1 SD Flow
	*β [CI*_*95%*_*]*	*β [CI*_*95%*_*]*
Worry	.14* [.10, .18]	.06* [.02, .10]
Negative emotion	.11* [.07, .14]	.007 [-.03, .04]
Depressive symptoms	.16* [.12, .19]	.02 [-.01, .06]
Anxious symptoms	.12* [.09, .16]	.01 [-.02, .05]
Loneliness	.18* [.14, .21]	.07* [.03, .11]
Unhealthy behaviors	.12* [.08, .16]	.05 [.003, .09]

Note

^*^p < .01. Estimates are from models that control for gender, age, sibling status, education, family income, dispositional optimism, intolerance of uncertainty, and satisfaction with life.

### Mindfulness

As predicted, mindfulness was a consistent predictor of overall well-being. With the exception of worry (weak association) and unhealthy behavior (positive association), participants who reported experiencing more mindfulness reported better well-being. However, in contrast to flow, mindfulness did not significantly moderate the association between quarantine length and any measure of well-being.

### Alternative analyses

Because of the majority of the sample was not yet in quarantine, we also ran models coding quarantine length as -0.5 = no quarantine (*n* = 3762), +0.5 = 14+ days of quarantine (*n* = 934). The results were nearly identical, except that flow no longer significantly moderated the associations between loneliness and quarantine length (just below significance) or unhealthy behavior and quarantine length, and mindfulness no longer showed a significant association with loneliness (just below significance).

## Discussion

This study provides a unique opportunity to understand the experiences of people at ground zero for a worldwide pandemic, nearly in real-time. As we write this paper in October 2020, COVID-19 has been nearly eradicated in China; however, the number of new cases in the U.S. continues to rise rapidly, and millions of people worldwide are currently in or will soon enter (or re-enter) some form of quarantine or isolation. As such, our findings provide timely guidance to those experiencing distress associated with quarantine conditions.

Specifically, the findings reveal three key insights about this unique and stressful experience, which may generalize to similar periods of open-ended uncertainty. First, people who had endured a longer quarantine reported poorer well-being by many definitions: more worry, more negative emotions, more depressive and anxious symptoms, more loneliness, and more unhealthy behaviors. Second, experiencing flow and mindfulness was associated with better well-being during the stressful circumstances in which people found themselves in China in February 2020, regardless of quarantine status or length. Comparing effect sizes in Table 1, flow seemed particularly associated with reduced loneliness, engagement in healthy behaviors, and avoidance of unhealthy behaviors, whereas mindfulness was more associated with fewer negative emotions and anxious symptoms—although mindfulness was also associated with greater loneliness and more unhealthy behavior. Flow and mindfulness were approximately equally associated with elevated positive emotions and reduced depressive symptoms.

Third, and perhaps most novel, flow—but not mindfulness—moderated the link between quarantine length and well-being deficits. In fact, people in a lengthy quarantine who reported higher-than-average flow experiences in the past week (i.e., +1 SD) were no worse off than people who were not yet quarantined on many measures of well-being, statistically speaking (see Table 2). Length of quarantine remained significantly associated with worry and loneliness among those high in flow (albeit a far weaker association compared to those low in flow), which suggests the possibility that worry and loneliness may be particularly stubborn forms of distress during quarantine. In contrast, mindfulness did not moderate any link between quarantine length or status and well-being.

Why did flow so consistently moderate what appear to be deleterious effects of quarantine length—and equally important, why did mindfulness not? In short, flow is a form of exquisite distraction, perfect for situations in which the primary goal is to make time pass more quickly [12, 46]. As a quarantine wears on, people may find that the tedium of isolation allows their worries to run wild, with little else to keep their mind occupied. If instead people can find activities that absorb their attention, the days feel shorter and the weeks, therefore, more tolerable. Of course, it is likely that not all forms of distraction are so consistently beneficial to well-being; for example, if a problem needs to be addressed or emotions processed, distraction may be detrimental to well-being in the long-run. However, flow is a particularly beneficial form of distraction, perhaps because it entails active engagement rather than avoidance or suppression.

Mindfulness, on the other hand, draws attention toward one’s present circumstances and internal states. As in daily life, our findings suggest that mindfulness is good for reducing distress in a given moment or on a given day during a widespread health crisis. However, mindfulness is nearly antithetical to distraction (in fact, a reverse-coded item on the scale used in this study is “I have been easily distracted”), and flow-inducing distractions may be the very best medicine to bolster well-being during a lengthy quarantine.

We would note that although we asked participants to reflect on their experiences of mindfulness and flow during the previous week, the measure likely tapped into both state and trait aspects of these variables. That is, a person who reported a high degree of mindfulness during the past week is likely also someone who is dispositionally mindful. We attempted to mitigate the influence of dispositional characteristics by controlling for conceptually-related individual differences, namely dispositional optimism, intolerance of uncertainty, and satisfaction with life. Nonetheless, our findings may also point to well-being advantages among those naturally disposed toward mindfulness and flow.

Although our study had a number of strengths, most notably the large sample size and the timely, highly significant real-world context, several weaknesses are worth noting. The study was cross-sectional, thus rendering our data incapable of testing causal hypotheses. We argue that our interpretation of the direction of effects is the most parsimonious, given our efforts to control for likely third variables and the well-established causal links between flow/mindfulness and well-being in other contexts. However, it is possible that some relationships are reciprocal, such that experiencing distress disrupts people’s ability to find flow and remain mindful, or that we failed to assess relevant third variables. Similarly, it is possible that people who were more distressed opted into quarantine (i.e., reverse causality) rather than quarantine conditions causing deterioration of well-being. We propose that the most parsimonious explanation is a deleterious effect of isolation and restricted movement, particularly given other research that found such effects in other pandemics [e.g., 2, 4] and in the current pandemic [in France; 5] and given the relatively strict policies regarding quarantine during that period in China, quite different from the United States and other countries with more lenient policies. That said, quarantine status was almost certainly a function of where our participants lived within China, thus introducing the possibility of third-variable explanations in those associations as well.

In addition, we would note that a large majority of our sample was not (yet) in quarantine at the time they completed the survey, although presumably all of our participants were keenly aware of the growing health crisis in their country and experienced significant disruptions to their lives. Ideally, we would have followed participants longitudinally as they passed in and out of quarantine restrictions, or at least had more even coverage across various lengths of quarantine. Furthermore, our sample was skewed toward relatively educated participants (nearly all had a Bachelor’s degree), given the nature of the data collection strategy.

Another unavoidable feature of our sample was the relatively short average length of quarantine (weeks rather than months). Our study cannot address the usefulness of flow and mindfulness across longer periods of distancing and isolation like those that are now the norm in the United States and other countries. On the other hand, our study is well-suited to address a particular type of isolation that remains common, namely the more extreme quarantine recommendations following potential exposure to someone with COVID-19 or a positive test for the virus. Our findings may be most helpful to people in these relatively short, time-limited, and highly restrictive conditions.

Finally, we were limited in the length of our survey by practical constraints, and thus we do not have information about the activities that most effectively or consistently promoted flow states. Other studies have found that although people seem capable of reporting on their flow states, they are less successful at identifying activities that are particularly flow-inducing [12]. Although flow activities are highly idiosyncratic, activities that provide the right level of challenge (not too hard, not too easy) and offer feedback on one’s progress are most reliable at creating flow states [6]. A key direction for future research is to more clearly identify the types of activities that work best for the most people, as well as examining the importance of person-activity fit, during periods of quarantine-like conditions.

Despite these limitations, this study offers a rare glimpse inside a community at the initial epicenter of a once-in-a-century pandemic. At the time the data were collected, Wuhan was unique in its strict restrictions and widespread lockdown. In the relatively brief time since then, the crisis has grown and spread exponentially, such that people across the world are experiencing conditions like those of our participants. Our findings provide a glimmer of hope for people struggling with the challenges of social distancing or even complete quarantine: The time may pass more quickly if you find your flow.
